# PReS-FINAL-2018: The P53 gene polymorphisms and juvenile idiopathic arthritis

**DOI:** 10.1186/1546-0096-11-S2-P31

**Published:** 2013-12-05

**Authors:** A Kozhevnikov, N Pozdeeva, M Konev, M Nikitin, V Larionova, G Novik, M Slyndev

**Affiliations:** 1 Saint-Petersburg State Pediatric Medical University, Russian Federation; 2 Hospital Department of Orthopedics and Rheumatology, Saint-Petersburg Research Pediatric Orthopedic Institute n. a. G.I. Turner, Russian Federation; 3 "Lumex ltd", Saint-Petersburg, Russian Federation

## Introduction

Juvenile idiopathic arthritis (JIA) is a multifactor chronic inflammatory joints disease that is characterized by long progressive course leading to the development of contractures and loss of joint function. Prediction of the likely outcomes and course of JIA is key approach of treatment. Currently, there are only several laboratory and radiological predictors of unfavorable prognosis of JIA. Reduced sensitivity of cells to apoptosis is one the possible mechanisms maintaining synovial inflammation in JIA. Polymorphisms *Arg72Pro 4exon*, *ins/del16bp 3intron*, *G/C 6intron *can determine activity of the *P53 *protein - the key protein of intrinsic apoptosis pathway (P.Dumont et al 2003; A.Sallivan et al, A.Ghosh et al 2004).

## Objectives

We evaluated role of P53 gene polymorphisms in the course and outcome of JIA.

## Methods

Clinical, serological, x-ray manifestations, ultrasound and MRI were analyzed in 126 children with JIA. 60 healthy children without family history of any autoimmune disease were controls. The *P53 *gene polymorphisms were detected by PCR-RFLP.

## Results

We haven't revealed significant differences in genotypes distributions of *Arg72Pro ex4*, *ins/del16bp in3 *and *G/C in6 *between children with JIA and controls. We identified significant difference of exon genotype *Arg72Pro *in girls who still persistently in ≪active disease≫ more 2 years compared with girls who achieved clinical remission (Arg/Arg - 36,7% vs 83,3%, Arg/Pro - 51,1% vs 16,7%, Pro/Pro - 12,2% vs 0%). 68% girls who achieved ≪inactive≫ oligoarthicular (OA) and polyarthicular (PA) were with homozygous genotype *Arg/Arg*. Girls with *Pro *allele genotypes (*Arg/Pro*, *Pro/Pro*) have the highest rates of probability of the ≪active disease≫ for OA (OR = 158,3, p = 0,0001) and PA (OR = 32,8, p = 0,005) compared with ERA (OR = 0,5, p = 0,8). Boys haven't any difference of exon genotype in JIA (p > 0,05, OR < 2,5). We received no information about influence of intronic polymorphisms on the JIA course. We haven't any difference of polymorphisms *P53 *gene in children with joints erosion and arthrosis.

Haplotypes */del-Arg/Arg-GG were significantly higher in girls with clinical remission. Girls who still maintain an ≪active≫ OA and PA course had haplotypes */del-Arg/Pro-G*, ins/del-Pro/Pro-GC, ins/ins-Pro/Pro-CC. Most girls with ins/del-Pro/Pro-GC, ins/ins-Pro/Pro-CC were with fibrous ankylosis.

## Conclusion

We suggest that *Pro *allele genotypes of the exon polymorphism *P53 *gene contribute to persistence of ≪active disease≫ in girls with oligo-, polyarthritis. Analysis *Arg72Pro 4exon*, *ins/del16bp 3intron*, *G/C 6intron *polymorphisms *P53 *gene can be used in a comprehensive assessment unfavorable risk of *JIA course *in girls.

## Disclosure of interest

None declared.

